# C-Reactive Protein in Stable Cystic Fibrosis: An Additional Indicator of Clinical Disease Activity and Risk of Future Pulmonary Exacerbations

**DOI:** 10.4172/2161-105X.1000375

**Published:** 2016-10-14

**Authors:** Elias Matouk, Dao Nguyen, Andrea Benedetti, Joanie Bernier, James Gruber, Jennifer Landry, Simon Rousseau, Heather G Ahlgren, Larry C Lands, Gabriella Wojewodka, Danuta Radzioch

**Affiliations:** 1 Adult Cystic Fibrosis Clinic, Montreal Chest Institute, McGill University, Canada; 2 Department of Human Genetics, Respiratory Division, McGill University, Canada; 3 McGill University Health Center Research Institute, Canada; 4 Department of Medicine, McGill University, Canada; 5 Department of Epidemiology, Biostatistics and Occupational Health, Montreal Chest Institute, McGill University Health Center, Canada; 6 Meakins-Christie Laboratories, Montreal Chest Institute, McGill University Health Centre, Canada; 7 Division of Pediatric Respiratory Medicine, Montreal Children’s Hospital, Canada

**Keywords:** Systemic biomarkers, Inflammation, Clinical scoring, Clinical disease severity, Quality of life, Chronic pulmonary infections, Lung function

## Abstract

**Introduction:**

In stable adult cystic fibrosis (CF) patients, we assessed the role of baseline high sensitivity C-reactive protein (hs-CRP) on CF clinical variables and frequency of intravenous (IV) treated pulmonary exacerbations (PExs) 1-year post-baseline.

**Methods:**

We recruited 51 clinically stable CF patients from our Adult CF Center. We incorporated collected parameters into Matouk CF clinical score and CF questionnaire-revised quality of life score (QOL). We used the clinical minus complications subscores as a clinical disease activity score (CDAS). We dichotomized our patients according to the cohort median baseline hs-CRP of 5.2 mg/L.

**Results:**

Patients in the high hs-CRP group (≥ 5.2 mg/L) demonstrated worse CDAS (r=0.67, p=0.0001) and QOL scores (r=0.57, p=0.0017) at a given FEV_1_% predicted. In both hs-CRP groups, prior-year IV-treated PExs and baseline CDASs were significant predictors of future IV-treated PExs. Interestingly, the association between baseline CDAS and future PExs frequency was more robust in the high compared to the low hs-CRP group (r=−0.88, p<0.0001, r=−0.48, p=0.017, respectively) with a steeper regression slope (p=0.001). In addition, a significant interaction was demonstrated between elevated baseline hs-CRP levels and CDASs for the prediction of increased risk of future PExs (p=0.02). This interaction provided an additional indicator of clinical disease activity and added another dimension to the prior year PExs frequency phenotype to identify patients at increased risk for future PExs.

**Conclusion:**

Stable CF patients with elevated baseline hs-CRP (≥ 5.2 mg/L) demonstrated worse clinical disease activity and QOL scores at a given level of disease severity (FEV_1_% predicted). Elevated baseline hs-CRP values combined with clinical disease activity scores are associated with increased risk for future IV-treated PExs even in those with mild clinical disease activity scores.

## Introduction

Cystic fibrosis (CF) lung disease is characterized by a chronic bacterial infection associated with a persistent, exaggerated, and ineffective neutrophil-mediated inflammation. Both infection and inflammation lead to permanent structural damage of the airways that progress to respiratory failure and death [1].

Pulmonary exacerbations (PExs) requiring intravenous (IV) antibiotic treatment represent important clinical events in the course of CF disease. An increased frequency of PExs significantly contributes to morbidity, the rate of decline of forced expiratory volume in 1 second, and mortality [2–4]. Systemic markers of inflammation, C-reactive protein (CRP), calprotectin, and vascular endothelial growth factor, are biomarkers that most consistently correlate with acute PExs of CF. These biomarkers increase at the clinical onset of such events and decrease with effective treatment [5].

Three previous prospective studies used systemic biomarkers at the end of CF PExs treatment for predicting the time until the next exacerbation [5–7].

Wojewodka et al. [7] studied a cohort of 52 adult patients with CF. They demonstrated that stable patients who developed PExs over a 1-year follow-up had worse baseline disease severity (based on spirometry), worse baseline Clinical and Complications subscores based on the Matouk CF Disease Score, and worse self-reported quality of life (QOL) scores. Interestingly, this was associated with higher inflammatory markers, namely interleukin-6 and interleukin-10. In their study, there was a trend (not significant) for high CRP levels to be associated with a high risk of PExs. Reid et al. [8] recently studied 57 adult patients with CF. They showed that in stable patients, serum calprotectin levels at baseline predicted future exacerbations in terms of a shorter time to the next exacerbation. Additionally, there was a decline in lung function, particularly forced vital capacity, over a 1-year follow-up. A much weaker association was noted with baseline CRP, with no effect on a decline in lung function.

Three studies in patients with CF identified prior intravenously (IV)-treated PExs as a significant hazard for future IV-treated PExs [9–11]. A more recent multicenter study showed that the number of prior-year IV-treated PExs of CF was a primary independent risk factor for future IV-treated PExs over 24-weeks follow-up [12]. This was the case even after adjustment with significant demographic and clinical covariates. There were no systemic or sputum biomarkers included in the covariates.

In the present study, we aimed to further examine the role of baseline high sensitivity CRP (hs-CRP) levels on clinical CF disease variables. We hypothesized that elevated baseline hs-CRP levels in stable adult patients with CF are associated with the following: 1) worse disease severity (as measured by FEV_1_% predicted), worse clinical and complications subscores (as an index of clinical disease activity) and QOL scores, and increased frequency of IV-treated PExs 1-year prior and post-baseline; and 2) worse clinical disease activity for the same level of disease severity. We also wished to explore the role of baseline hs-CRP levels on the ability of predictor variables, such as baseline FEV_1_% predicted, clinical and complications subscores, and the number of IV-treated PExs 1-year prior to the beginning of the study (baseline; prior-year PExs), to determine the frequency of IV-treated PExs 1-year post-baseline (PExs 1-year post-baseline).

## Methods

In this observational cohort study, we enrolled 51 clinically stable patients who were followed at the Adult CF Clinic at the Montreal Chest Institute and prospectively followed them for 12 months. Clinical stability was defined as the absence of PExs requiring IV or oral antibiotic therapy in the preceding 4 weeks. There is no standard approach to prospectively define an exacerbation or to assess its severity. We used The European Consensus Group’s view to define PExs as the need for additional antibiotic treatment as indicated by a recent change in at least two of the following: a change in sputum volume or color; increased coughing; increased dyspnea; increased malaise, fatigue or lethargy; anorexia or weight loss; and a decrease in pulmonary function by ≥ 10% or radiographic changes [13]. These features have been used to define PExs in a national treatment guideline [14]. In this study, we defined IV-treated PExs as those with severe enough symptoms and signs affecting patients’ activities [15]. We also extracted from the patients’ medical records the number of IV-treated PExs 1-year prior to baseline.

All enrolled patients gave written consent to participate in the study. The study was approved by the Ethics Review Board of the McGill University Health Centre.

### Clinical data, clinical scoring, and plasma hs-CRP measurement

Demographic and clinical information, including patient-reported symptoms, were obtained at the initial visit and subsequent regular visits and those for PExs. Spirometry was performed according to standards that were established by the American Thoracic Society [16]. Microbiology data were obtained from routine sputum cultures at the McGill University Health Centre microbiology laboratory. Chest radiographs were scored according to the modified Brasfield scoring system. These data were incorporated into the Matouk CF score, which was previously described and validated [7,17,18]. Briefly, the total score comprises four subscores of clinical, pulmonary function (PFT), chest radiography (CXR), and complications subscores. Healthier patients have higher scores for clinical, PFT, and CXR subscores and lower values for the complications subscore. The Total Score=(Clinical+PFT+CXR subscores)–(the complications subscore). A full description of the scoring method is shown in supplementary file.

We also used the clinical minus complications subscore as a clinical disease activity score (CDAS). This score encompasses patient-reported key symptoms, physician-documented clinical signs, and CF-related complications adding to CF disease activity. The complications subscore includes: 1) The frequency of prior-year IV-treated PExs — A five-point deduction is allowed if the patient had an exacerbation over the past 3 months. A two-point deduction is allowed if the patient had an exacerbation over the past 12 months. A maximum deduction of seven points is allowed. The patient regains his deducted points in the absence of exacerbations over the respective periods. 2) The severity of hypoxemia, presence of hypercapnea, previous intubation. 3) The frequency and severity of hemoptysis. 4) The frequency of pneumothorax. 5) Cor pulmonale, right heart failure and 6) Pulmonary resection surgery. A lower clinical minus complications subscore denotes a worse CDAS.

High sensitivity CRP levels were measured in plasma samples by the extended range turbidimetric method: (SYNCHRON^®^ System(s) CRPH reagent; Beckman Coulter Inc., Brea, CA, USA). This hs-CRP test has an analytical range of 0.20 mg/L to 80.00 mg/L and a cut point of 5 mg/L as acute phase reactant. Analytical sensitivity can be defined as the lowest measurable concentration which can be distinguished from zero with 95% confidence is 0.11 mg/L. Functional sensitivity ≤ 0.09 mg/L (at Coefficient of Variance of 19.1%). Manufacturer’s Coefficient of Variance: 4.9% at 0.21 mg/L, 2.5% at 1.05 mg/L, 0.92% at 2.99 mg/L, 1.53% at 10 mg/L, 1.68% at 59.38 mg/L. Current Instrument Coefficient of Variance (in-house): 1.02% at 5.5 mg/L, 1.27% at 27 mg/L.

### Patient-reported QOL

The QOL evaluation was recorded at baseline (n=51) by a self-administered questionnaire using the CF questionnaire-revised (CFQ-R). The CFQ-R comprises 50 items associated with three symptom sores (weight, respiratory function, and digestion) and nine QOL domains (physical, vitality, emotional, eating disturbances, treatment burden, health perceptions, body image, social functioning, and role/school functioning) [19]. We also included a total score as the sum of the scores for all the items in the questionnaire. Higher scores reflect healthier disease status.

### Statistical analysis

Patients were dichotomized into two groups based on the median baseline plasma hs-CRP values of the cohort (low: <5.2 mg/L; high: ≥ 5.2 mg/L). The Student’s t-test or Mann-Whitney U test was used for comparison of means of all disease variables between the low and high hs-CRP groups. Fisher’s exact test (two-tailed) was used to assess the statistical significance of the association between certain CF-related complications, medications, genotype, and the two hs-CRP groups. Pearson correlations were used to estimate the various associations of FEV_1_% predicted, CDAS, QOL items, and frequency of PExs in the two hs-CRP groups. Analysis of covariance was used to assess the statistical significance of differences between regression slopes. We then used Poisson regression models to estimate the interactions between baseline continuous hs-CRP values, FEV_1_% predicted, CDAS, and the frequency of prior-year PExs for prediction of the frequency of PExs 1-year post-baseline. Statistical significance was set at p values <0.05. Data were analyzed using SAS version 9.4 statistical software (Cary, NC, USA).

## Results

### Patient cohort characteristics

A total of 51 patients were enrolled in this study and followed up for 12 months. A total of 53% of patients were female and the mean age was 33 years (SD 13.6 years). The mean FEV_1_% predicted was 62.6% (SD 28.27), representing a moderate level of disease severity. The mean body mass index was 21.9 kg/m^2^ (SD 4.16), representing adequate nutritional status [20]. The mean hs-CRP level was 7.2 mg/L (SD 6.7, range: 0.3 mg/L to 26.4 mg/L), with a median of 5.2 mg/L (interquartile range: 1.8 mg/L to 9.2 mg/L).

### Patients’ characteristics between the two groups according to baseline hs-CRP levels

We first performed a cross-sectional analysis to compare the characteristics of patients who were dichotomized into two groups according to their baseline hs-CRP levels measured during a stable clinical state (Table 1). Although such categorization may lead to loss of information compared with using continuous hs-CRP values, the use of cut points is clinically useful in evaluating their respective associations with indices of clinical disease severity and activity [21].

Patients with high hs-CRP levels had significantly lower FEV_1_% and FVC% predicted and worse CDASs than those with low hs-CRP levels (Table 1). The total QOL score, predominantly represented by the physical, vitality, health perceptions, body image, role functioning, and social domains, was also lower in patients with high hs-CRP levels than in those with low hs-CRP levels. However, there was only a trend (not significant) towards lower respiratory symptoms scores in patients with high hs-CRP levels. This finding might be secondary to the chronic nature of the symptoms captured in the CFQ-R respiratory score that are pervasive among patients with different degrees of disease severity.

We demonstrated a significant correlation between bacteriological score and hs-CRP levels (r=−0.56, p<0.0001). However, the sputum microbiology of the two hs-CRP groups was significantly different. Notably, 93% of patients in the high CRP group were colonized with *Pseudomonas aeruginosa* (74% mucoid *P. aeruginosa*, 11% multidrug resistant *P. aeruginosa*, 7% non-mucoid *P. aeruginosa*) and only 7% were colonized with *Staphylococcus aureus*. In contrast, 53% of patients in the low CRP group were colonized with *P. aeruginosa* (21% mucoid *P. aeruginosa*, 8% multidrug resistant *P. aeruginosa*, 17% non-mucoid *P. aeruginosa*), 33% with *S. aureus* and 21% with normal flora. Interestingly, all patients with baseline hs-CRP>10 mg/L, accounting for 22% of the study cohort, were colonized with mucoid *P. aeruginosa* or multidrug resistant *P. aeruginosa*.

Also, the absolute neutrophil count, although within the normal limits in the 2 hs-CRP groups, was significantly more elevated in the high versus low hs-CRP group.

Importantly, patients in the high hs-CRP group experienced more PExs events in the year preceding and following the initial baseline visit (Table 1).

There were no significant differences in CF-related conditions (insulin-dependent CF-related diabetes, allergic bronchopulmonary aspergillosis, asthma and asthma-like symptoms), medications, and cystic fibrosis transmembrane regulator (CFTR) genotype between the two hs-CRP groups. There was a trend (not statistically significant) for Inhaled corticosteroids to be used more frequently in the low hs-CRP group compared to the higher group. Interestingly, in our study cohort, asthma and asthma-like symptoms were observed in 50% of the patients. Notably, 10 patients (37%) in the high hs-CRP group compared to 15 patients (62.2%) in the lower hs-CRP group (p=0.09) (Table 2).

### Role of dichotomized baseline hs-CRP levels on CDASs and QOL scores for a given FEV_1_% predicted

**Figure 1:**
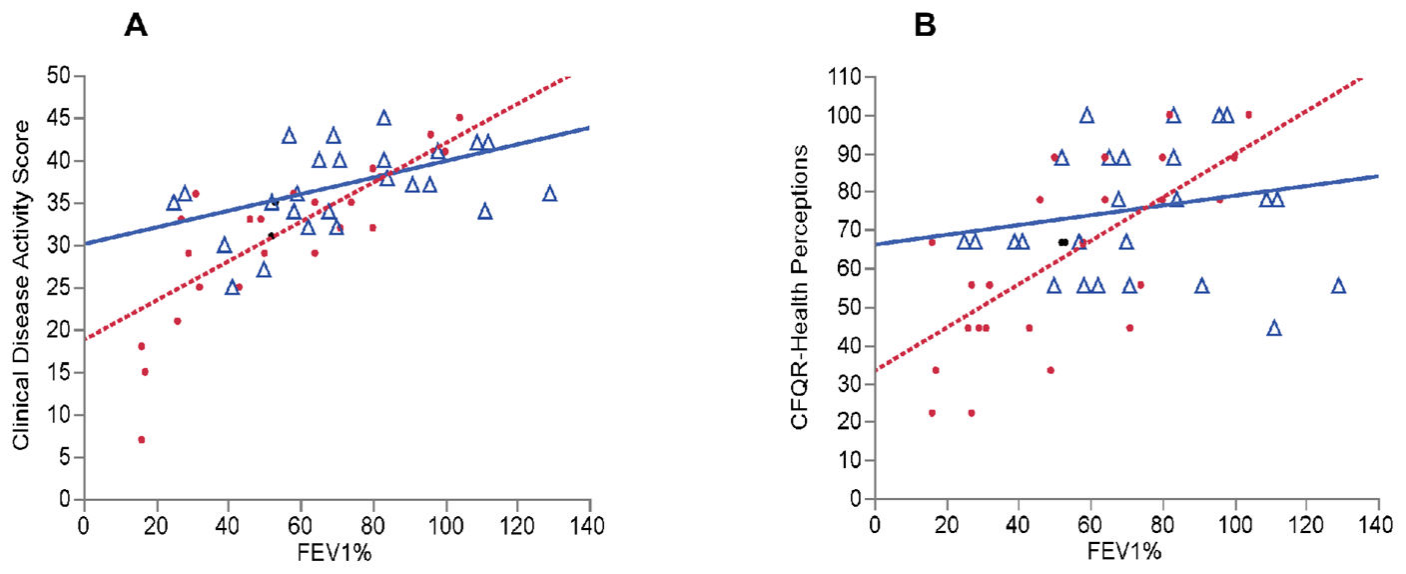
A) Correlations between the baseline CDAS and frequency of IV-treated PExs 1 year post baseline, dichotomized by CRP groups. △ indicates CRP levels <5.2 mg/L (r=−0.48, p=0.017); ● indicates CRP levels ≥ 5.2 mg/L (r=−0.88, p=<0.0001). The value p=0.001 indicates significance between regression slopes. B) Correlations between the frequency of prior-year and 1 year post-baseline IV-treated PExs, dichotomized by CRP groups. △ indicates CRP levels <5.2 mg/L (r=0.52, p=0.0089). ● indicates CRP levels ≥ 5.2 mg/L (r=0.77, p=<0.0001).

Because the low and high hs-CRP groups showed significant differences in their characteristics, we hypothesized that they represent patient populations with different relationships between lung function and clinical disease activity variables. We evaluated the role of baseline hs-CRP on CDAS and QOL scores at a given level of disease severity. We first observed that FEV_1_% predicted and the CDAS showed a significant correlation in the whole cohort (r=0.68, p<0.0001). However, this correlation was significantly more robust and showed a steeper slope in the high hs-CRP group compared with the low hs-CRP group (Figure 1A). Patients in the high hs-CRP group had lower CDASs at a given level of FEV_1_% predicted and a greater decline in the CDAS for a given decrease in FEV_1_% predicted than those in the low hs-CRP group.

We also observed a significant correlation between FEV_1_% predicted and the Health perceptions QOL domain in the entire patient cohort (r=0.5, p=0.0002). However, the correlation was significant only in the high versus low hs-CRP group (r=0.65, p=0.0002; r=0.17, p=0.1 respectively) and demonstrated a steeper slope (p=0.03) (Figure 1B).

We did not observe a significant correlation of FEV_1_% predicted with the respiratory symptoms scores.

**Figure 2:**
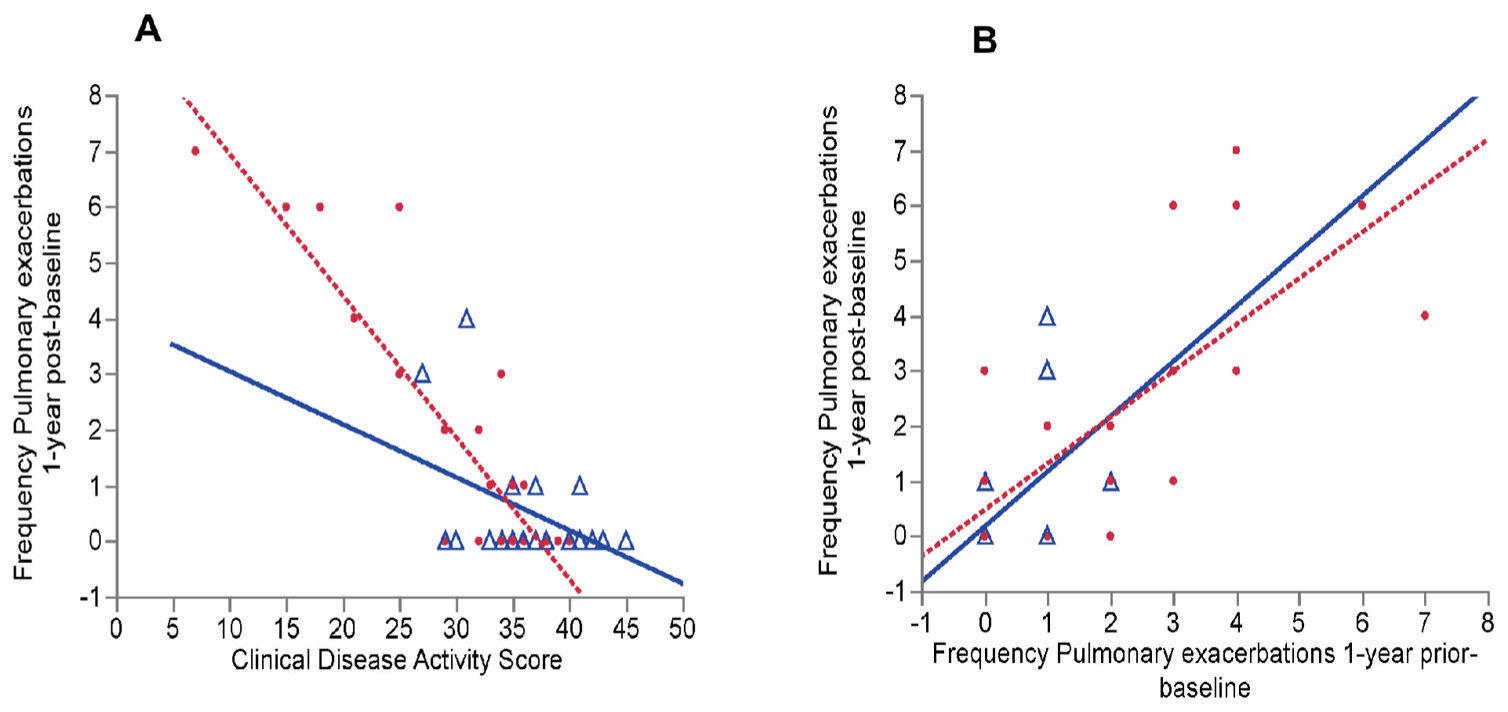
A) Correlations between FEV1% predicted and the CDAS dichotomized by CRP groups. △ indicates CRP levels <5.2mg/L (r=0.52, p=0.009); ● indicates CRP levels ≥ 5.2 mg/L (r=0.67, p=0.0001). The value p=0.03 indicates significance between regression slopes. B) Correlations between FEV1% predicted and the Health perceptions QOL domain dichotomized by CRP groups. △ indicates CRP levels <5.2 mg/L (r=0.17, p=0.1). ● indicates CRP levels ≥ 5.2 mg/L (r=0.65, p=0.0002). The value p=0.03 indicates significance between regression slopes.

Taken together, these results suggested that patients in the high hs-CRP group had a more clinically active disease as documented by physician and perceived by patients compared to those in the low hs-CRP group, despite similar lung function measurements (Figure 2).

### Role of dichotomized baseline hs-CRP levels on predictor variables for future PExs

We hypothesized that the two hs-CRP groups represent patient populations with different associations of predictor variables (FEV_1_% predicted, CDAS, and frequency of prior-year PExs) for the frequency of future PExs.

We expected that the risk of PExs is greater as lung function declines. The correlation between baseline FEV_1_% predicted and the frequency of PExs 1-year post-baseline was significant in the whole cohort (r=−0.54, p<0.0001). When dichotomized, we observed a significant correlation only in the high versus low hs-CRP group (r=−0.59, p=0.001; r=−0.301, p=0.15, respectively), with a steeper slope in the high hs-CRP group (p=0.008). However, the correlation was no longer significant when adjusted for the CDAS and prior-year PExs (r=0.04).

The correlation between the CDAS and the frequency of PExs 1-year post-baseline was significant in the entire patient cohort (r=−0.82, p<0.0001). However, this correlation was more robust in the high hs-CRP group compared with the low hs-CRP group, with a steeper slope in the high hs-CRP group (p=0.001) (Figure 1A). This correlation was still significant after adjusting for FEV_1_% predicted and prior-year PExs (r=−0.72).

The correlation between the frequency of prior-year PExs and the frequency of PExs 1-year post-baseline was significant in the whole cohort (r=0.77, p<0.0001), as well as in the high and low hs-CRP groups (Figure 1B). There was no significant change in this correlation after adjusting for FEV_1_% predicted and CDAS (r=0.48) (Figure 1).

### Poisson regression analysis of predictor variables for the frequency of future PExs

We then performed Poisson regression analysis to estimate the interactions of all predictor variables (CDAS, frequency of prior-year PExs, FEV_1_% predicted, continuous hs-CRP values) for the rate of future PExs. We first included all predictor variables in the model (Table 3). Each unit increase in CDAS was expected to decrease the frequency of future PExs by a factor of 0.93 (7.5% decrease), while the other variables were constant. Each unit increase in the frequency of prior-year PExs was expected to increase the frequency of future PExs by a factor of 1.33 (33% increase), while the other variables were constant. FEV_1_% predicted and continuous hs-CRP values did not reach statistical significance after adjusting for the other predictors.

We then performed Poisson regression analysis selectively with each predictor variable and its interaction with continuous hs-CRP values.

For each unit increase in CDAS, the expected risk ratio was 0.90 (95% CI: 0.88–0.92) (i.e., the rate of PExs was expected to decrease by 10%). There was a significant interaction between continuous hs-CRP values and CDAS (p=0.02). Therefore, in a subject with an hs-CRP value of 1.8 mg/L (25^th^ percentile), for each unit increase in CDAS, the rate of PExs was expected to decrease by 17%. For a subject with an hs-CRP of 5.2 mg/L, the rate of exacerbations was expected to decrease by 15%. For a subject with an hs-CRP value of 9.2 mg/L (75^th^ percentile), the rate of exacerbations was expected to decrease by 13%. Therefore, the strength of the association between a better CDAS and the frequency of future PExs slightly decreases as hs-CRP levels increase.

The frequency of prior-year IV-treated PExs was also predictive of the frequency of IV-treated PExs 1-year post-baseline. For each additional prior PEx, the rate of future PExs was expected to increase by 48%, (risk ratio 1.52 (95% CI: 1.35–1.71)), with no significant interaction with hs-CRP levels (p=0.19).

Baseline FEV_1_% predicted was predictive of the rate of future PExs. For each unit increase in FEV_1_% predicted, the exacerbation rate decreased by 4% (95% CI: 2–6). However, there was no effect modification by interaction with continuous hs-CRP values (p=0.29).

Baseline hs-CRP levels were predictive of the rate of PExs. Each unit increase in baseline hs-CRP levels was associated with an increased risk of the rate of exacerbations by 9% (risk ratio per unit increase in CRP level: 1.09; (95% CI: 1.05–1.14). After adjustment for FEV_1_% predicted, each unit increase in hs-CRP level was expected to increase the rate of exacerbations by 4%. However, this effect was not significant (p=0.11).

## Discussion

The present study provided important findings regarding the role of baseline hs-CRP levels in clinically stable adult patients with CF. We found significant cross-sectional differences in CF disease characteristics and outcomes in the patient cohort, which was dichotomized according to baseline hs-CRP values using the median cohort value as a cut-off level (5.2 mg/L). Patients in the high hs-CRP group demonstrated lower baseline FEV_1_% predicted, worse baseline CDASs and QOL scores, and a higher frequency of prior-year and 1-year post-baseline PExs compared with those in the low hs-CRP group. Patients in the high hs-CRP group also had worse CDAS and QOL scores for a given level of disease severity (FEV_1_% predicted) and a steeper slope of these variables for a given change in FEV_1_% predicted compared with those in the low CRP group. In both hs-CRP groups, prior-year PExs were a significant predictor of future PExs, with no interaction with hs-CRP values. Although baseline CDASs were a significant predictor for future PExs in the whole cohort, this relationship was more robust in the high compared to the low hs-CRP group and with a steeper regression slope. Furthermore, a significant interaction was observed between the CDASs and hs-CRP levels for the prediction of future PExs, thereby providing an additional indicator of clinical disease activity.

Additionally, we found a different distribution in sputum microbiology between the two hs-CRP groups. There was a predominance of mucoid *P. aeruginosa* in the high hs-CRP group and a predominance of *S. aureus* and normal flora in the low hs-CRP group. However, not all patients with predominant mucoid *P. aeruginosa* colonization had elevated baseline hs-CRP levels, while some patients with predominant *S. aureus* colonization had elevated hs-CRP levels. These findings may be partly explained by the heterogeneity of the airway bacterial infections and the divergent host inflammatory response they can elicit.

In view of the small number of patients, the contribution of associated diseases relevant to CF was not specifically addressed. Interestingly, there was a trend (not significant) for patients in the low CRP group to demonstrate more asthma symptoms compared to the high CRP group. Our findings are in agreement with the previously reported asthma and asthma-like symptoms in 40% to 70% of CF patients [22,23]. However, the term cystic fibrosis asthma is usually coined to describe patients with cystic fibrosis who have episodes of acute airway obstruction reversed by bronchodilators, a strong family history of asthma, or evidence of atopy [24–26]. According to these criteria, about 20% of patients with cystic fibrosis have asthma (about twice the expected prevalence) [25]. Further studies are needed to evaluate the natural history of CF asthma phenotype compared to CF patients who develop asthma-like symptoms during the course of their disease.

The cross-sectional differences between the two groups in our study suggested that stable patients with CF, even those with mild elevation in baseline hs-CRP levels, may be significantly associated with different clinical variables of CF. In fact, the median hs-CRP level in the high hs-CRP group was 8.8 mg/L (interquartile range: 7.2 mg/L to 17.8 mg/L), while the median of 0.8 mg/L and 99^th^ percentile of 10 mg/L were reported in healthy volunteers [27]. Additionally, 59% of these patients showed an a hs-CRP value between 5 mg/L and 10 mg/L. In the low hs-CRP group (median: 1.75 mg/L; interquartile range: 0.47 mg/L to 3.42 mg/L), 42% of patients had hs-CRP levels <1 mg/L, which is close to the reported median in healthy volunteers, and 72% had hs-CRP levels <3 mg/L (cut point set for patients with chronic obstructive pulmonary disease as a predictor of future outcomes) [28,29].

Previous studies have demonstrated an inverse correlation between hs-CRP levels at baseline and FEV_1_% predicted [30–33]. However, in contrast to our study, the directionality of the relationship was not determined [30]. None of these previous studies examined the role of baseline hs-CRP levels on indices of clinical disease activity, QOL items, and the frequency of future PExs at a given level of disease severity (FEV_1_% predicted).

In the whole cohort, we observed that lower FEV_1_% predicted was associated with a higher rate of PExs 1-year post-baseline. However, this association was only significant in the high hs-CRP group and was no longer significant after adjusting for the CDAS and the rate of prioryear PExs.

In both hs-CRP groups, prior-year pulmonary exacerbations and baseline CDAS were significant predictors of future pulmonary exacerbations. Interestingly, the association between CDASs and future pulmonary exacerbations frequency was more robust in the high compared to the low hs-CRP group. In addition, a significant interaction was demonstrated between baseline hs-CRP levels and CDASs for the prediction of future PExs. This interaction suggested that the potential beneficial role of better CDASs in predicting a decreased risk for future PExs is less efficient at high hs-CRP values. As previously mentioned, the CDAS includes the frequency of prior-year PExs (up to a maximum of 7 points deduction over the preceding 12 months), as well as the effect of these exacerbations on the items of the clinical and complications subscores. Our results suggest that a state of heightened and persistent low-grade systemic inflammation, as observed by an elevation in baseline hs-CRP levels, may contribute to increased susceptibility for future PExs. Given the hs-CRP plasma half-life of 19 hours, the sole determinant of persistent elevation in circulating hs-CRP concentrations is the intensity and persistence of the pathological process(es) stimulating its production [22]. Although the molecular and biological mechanisms underlying this systemic inflammatory state are unclear, our findings may add to our understanding of the relationships between clinical disease severity/activity and inflammation.

Our results add a further dimension to the contribution of the prior-year PExs phenotype for identifying patients at increased risk for future PExs. They may help in evaluating this predictor variable within the context of the overall clinical picture and interaction with a systemic inflammatory biomarker, such as hs-CRP. Our findings resemble those observed by Thomsen et al. [21]. They found that in patients with chronic obstructive pulmonary disease, elevated levels of inflammatory biomarkers (CRP, fibrinogen, and leukocyte count) were associated with an increased risk of PExs, even in those with mild chronic obstructive pulmonary disease.

This enhanced risk stratification may also help in identifying patients who could benefit most from more intensive therapy targeting the vicious circle of infection and inflammation. Interestingly, Ratjen et al. [34] evaluated oral azithromycin in pediatric patients with CF who were uninfected with *P. aeruginosa*. They found a significant reduction in systemic inflammatory markers (hs-CRP, calprotectin, serum amyloid A), associated with improvement in FEV_1_ (L) and FEV_1_% predicted within 28 days of treatment. Additionally, Sullivan et al. [35] reported a retrospective analysis of the effects of lumacaftor and ivacaftor on inflammatory biomarkers in patients homozygous for the F508del-CFTR mutation. They observed improvement in FEV_1_% predicted associated with a reduction in calprotectin, hs-CRP, IgG, interleukin-8 levels and Neutrophil cell count. In that study, the reduction of IV-treated PExs rates was more pronounced compared to the modest improvement in FEV_1_% predicted. Their findings support the effect of a CFTR modulator on CF-mediated inflammation. These studies highlight the beneficial effect of modulating a heightened inflammatory state in patients with CF and underscore the potential clinical significance of our results.

The present study has several limitations. As a single-center, observational, cohort study, its modest number of patients limits its statistical power and generalizability. We also evaluated the contribution of a single baseline measurement of hs-CRP to assess its associations with lung function and clinical disease activity in a cross-sectional manner. Therefore, we can only infer an association, without any insight into the causal relationships involved. We also need to exclude the possibility that elevated hs-CRP levels are not the result of another pulmonary or extra-pulmonary pathology, which is infectious or inflammatory.

Larger, multicenter, prospective, longitudinal studies are required to evaluate the role of hs-CRP and other systemic biomarkers alone or in combination in tracking disease activity and progression.

## Conclusions

In conclusion, stable adult patients with CF with elevated baseline hs-CRP levels (≥ 5.2 mg/L) show not only lower baseline FEV_1_% predicted, but worse baseline clinical disease activity and QOL scores, and increased frequency of prior-year and 1-year post-baseline PExs. These patients also have worse CDASs and QOL scores at a given level of disease severity (FEV_1_% predicted). An elevated baseline hs-CRP level combined with CDAS is associated with an increased risk for future IV-treated PExs, even in those with a mild CDAS.

## Supplementary Material

supplementary file_scoring method

## Figures and Tables

**Table 1:** Patients’ characteristics according to baseline hs-CRP levels. Data are shown as mean (SD). The p values designate statistical difference between the two groups using the Student’s t-test or Mann-Whitney U test when values were not normally distributed. Significance was set at p<0.05.

Variables	hs-CRP <5.2 mg/L (N=24)	hs-CRP ≥ 5.2 mg/L (N=27)	P
	Mean (SD)	Mean (SD)	
Age last visit (years)	36.5 (15.2)	30.66 (11.9)	0.13
CRP mg/L (F/M)	2.1 (1.6)/1.8 (1.5)	11.6 (5.02)/11.9 (7.1)	0.68/0.86
FEV_1_% predicted	76.5 (28.1)	49.8 (22.5)	**0.0005**
FVC% predicted	94 (23.1)	66.62 (23.9)	**0.0001**
BMI (kg/m^2^)	22.6 (4.7)	21 (3.5)	0.182
Clinical subscore (0–50 points)	38.7 (4.1)	34.03 (3.6)	**0.0001**
Complications subscore (0–37 points)	1.2 (1.8)	3.77 (4.6)	**0.016**
PFT subscore (0–25 points)	19.08 (5.8)	14.22 (5.8)	**0.009**
CXR subscore (0–25 points)	18.5 (2.5)	14.8 (2)	**<0.0001**
Total score (0–100 points)	74.7 (12.6)	59.37 (13.5)	**0.0001**
Clinical-complications subscore†	37.4 (5.3)	30.3 (7.7)	**0.0005**
Bacteriological subscore (1–5 points)	3.4 (1.2)	2.11 (0.6)	**<0.0001**
CFQ-R domains and symptoms			
Weight	68 (33)	61.7 (41)	0.55
Respiratory	71 (19.4)	63.3 (12.2)	0.07
Digestion	81.5 (12.9)	75.3 (20.9)	0.21
Physical	82.4 (19)	59.7 (26.2)	**0.001**
Vitality	68.7 (14.1)	59.5 (14.7)	**0.028**
Emotion	84.7 (16)	78 (15.9)	0.14
Eating	92.8 (11.8)	88.8 (19.7)	0.39
treatment burden	70.3 (29.2)	63.7 (17.3)	0.32
health perceptions	75.9 (19.8)	61.3 (19.5)	**0.011**
body image	79.6 (21.6)	63.3 (26.4)	**0.02**
Social	80 (17)	68.72 (16.9)	**0.02**
Role	90.6 (13.7)	75.5 (21.9)	**0.005**
Total (0–1200 points)	946 (137)	819.3 (148.4)	**0.002**
NE (10^9^/L) NR: 1.6–7.7)	5.58 (2.1)	7.63 (2)	**0.001**
IV Antibiotics 1 year prior (N)	0.25 (0.53)	1.55 (2.04)	**0.003**
IV Antibiotics 1 year post (N)	0.41 (1.01)	1.77 (2.22)	**0.008**

CFQ-R: Cystic Fibrosis Questionnaire-Revised, domains and symptoms scores; NE: absolute neutrophil count; NR: Normal Range; N: number of pulmonary exacerbations requiring intravenous (IV) antibiotics;

† Clinical-Complications subscore was used as the clinical disease activity score (CDAS).

**Table 2:** Associated conditions, medications, genotype in the 2 hs-CRP groups.

Variables	CRP<5.2 mg/L	CRP ≥ 5.2 mg/L	P*
	(N=24)	(N=27)	
**Associated conditions**	**Number (%)**	**Number (%)**	
CFRD	3 (11%)	5 (18%)	0.7
ABPA, on prednisone	0	2 (7%)	1
Asthma & asthma like symptoms	15 (62%)	10 (37 %)	0.09
**Medications**			
ICS (inhaled corticosteroids)	15 (62%)	10 (37%)	0.09
**Prednisone, oral**	0	2 (7%)	0.49
Inhaled Tobi or aztreonam or Colistin	13 (54%)	18 (66%)	0.4
dornase alpha	12 (50%)	18 (66%)	0.26
Azithromycin	15 (62.5%)	23 (85%)	0.1
Ibuprofen	0	1 (3%)	1
**Genotype**			
ΔF508/ΔF508	10 (541%)	14 (51%)	0.57
ΔF508/other	10 (41%)	11 (40%)	1
other/other	4 (16%)	2 (4%)	0.4

CFRD: Cystic Fibrosis-Related Diabetes; ABPA: Allergic Bronchopulmonary Aspergillosis. ΔF508 indicates F508del-CFTR;

* Fisher’s exact test (two-tailed).

**Table 3:** Poisson regression estimates of all predictor variables for the frequency of PExs 1-year post-baseline.

Parameter	Rate ratio	P	95% CI
CDAS	**0.93**	**0.0006**	(0.88, 0.96)
Frequency prior year PExs*	**1.33**	**0.0014**	(1.12, 1.58)
FEV_1_% predicted	0.99	0.37	(0.97, 1.0)
CRP	0.95	0.13	(0.89, 1.0)

* Estimated via Poisson regression. CDAS: Clinical disease activity score, frequency of prior-year PExs, FEV_1_% predicted, and continuous CRP values were included as linear effects.

## Abbreviations

CF: Cystic Fibrosis
hs-CRP: High sensitivity C-Reactive Protein
PEx: Pulmonary Exacerbation
PExs: Pulmonary Exacerbations
CFQ-R: Cystic Fibrosis Questionnaire-Revised
QOL: Quality of Life
FEV_1_% predicted: Forced Expiratory Volume in one Second Percent Predicted
CDAS: Clinical Disease Activity Score
IV: Intravenous
Prior-year PExs: IV-treated Pulmonary Exacerbations 1-year Prior to Baseline
PExs 1-year post-baseline: IV-treated Pulmonary Exacerbations 1-year Post Baseline
CFRD: Cystic Fibrosis Related Diabetes
ABPA: Allergic Bronchopulmonary Aspergillosis
CFTR: Cystic Fibrosis Transmembrane Regulator Protein
ΔF508: F508del-CFTR

## References

[1] Cantin AM, Hartl D, Konstan MW, Chmiel JF (2015). Inflammation in cystic fibrosis lung disease: Pathogenesis and therapy. J Cyst Fibros.

[2] Sanders DB, Hoffman LR, Emerson J, Gibson RL, Rosenfeld M (2010). Return of FEV1 after pulmonary exacerbation in children with cystic fibrosis. Pediatr Pulmonol.

[3] de Boer K, Vandemheen KL, Tullis E, Doucette S, Fergusson D (2014). 1) Exacerbation frequency and clinical outcomes in adult patients with cystic fibrosis. Thorax.

[4] Liou TG, Adler FR, Fitzsimmons SC, Cahill BC, Hibbs JR (2001). Predictive 5-year survivorship model of cystic fibrosis. Am J Epidemiol.

[5] Gray RD, Imrie M, Boyd AC, Porteous D, Innes JA (2010). Sputum and serum calprotectin are useful biomarkers during CF exacerbation. J Cyst Fibros.

[6] Sequeiros IM, Jarad N (2012). Factors associated with a shorter time until the next pulmonary exacerbation in adult patients with cystic fibrosis. Chron Respir Dis.

[7] Wojewodka G, de Sanctis JB, Bernier J, Bérubé J, Ahlgren HG (2014). Candidate markers associated with the probability of future pulmonary exacerbations in cystic fibrosis patients. PLoS One.

[8] Reid P, McAllister DA, Innes JA, Porteous D, AndreGreening AP (2015). Measurement of Serum Calprotectin in Stable Patients Predicts Exacerbation and Lung Function Decline in Cystic Fibrosis. Am J Respir Crit Care Med.

[9] Block JK, Vandemheen KL, Tullis E, Fergusson D, Doucette S (2006). Predictors of pulmonary exacerbations in patients with cystic fibrosis infected with multi-resistant bacteria. Thorax.

[10] Vandevanter DR, Yegin A, Morgan WJ, Millar SJ, Pasta DJ (2011). Design and powering of cystic fibrosis clinical trials using pulmonary exacerbation as an efficacy endpoint. J Cyst Fibros.

[11] VanDevanter DR, Pasta DJ, Konstan MW (2015). Treatment and demographic factors affecting time to next pulmonary exacerbation in cystic fibrosis. J Cyst Fibros.

[12] VanDevanter DR, Morris NJ, Konstan MW (2016). IV-treated pulmonary exacerbations in the prior year: An important independent risk factor for future pulmonary exacerbation in cystic fibrosis. J Cyst Fibros.

[13] Bilton D, Canny G, Conway S, Dumcius S, Hjelte L (2011). Pulmonary exacerbation: towards a definition for use in clinical trials. Report from the EuroCareCF Working Group on outcome parameters in clinical trials. J Cyst Fibros.

[14] Flume PA, Mogayzel PJ, Robinson KA, Goss CH, Rosenblatt RL (2009). Cystic fibrosis pulmonary guidelines: treatment of pulmonary exacerbations. Am J Respir Crit Care Med.

[15] Abbott J, Holt A, Hart A, Morton AM, MacDougall L (2009). What defines a pulmonary exacerbation? The perceptions of adults with cystic fibrosis. J Cyst Fibros.

[16] (1987). Standardization of spirometry-1987 update. Statement of the American Thoracic Society. Am Rev Respir Dis.

[17] Matouk E, Ghezzo RH, Gruber J, Hidvegi R, Gray-Donald C (1997). Internal consistency reliability and predictive validity of a modified N. Huang clinical scoring system in adult cystic fibrosis patients. Eur Respir J.

[18] Matouk E, Ghezzo RH, Gruber J, Hidvegi R, Gray-Donald K (1999). Construct and longitudinal validity of a modified Huang clinical scoring system in adult cystic fibrosis patients. Eur Respir J.

[19] Quittner AL, Sweeny S, Watrous M, Munzenberger P, Bearss K (2000). Translation and linguistic validation of a disease-specific quality of life measure for cystic fibrosis. J Pediatr Psychol.

[20] (2012). The Canadian Cystic Fibrosis Registry.

[21] Thomsen M, Ingebrigtsen TS, Marott JL, Dahl M, Lange P (2013). Inflammatory Biomarkers and Exacerbations in Chronic Obstructive Pulmonary Disease. JAMA.

[22] Sanchez IPR, Powell RE, Pasterkamp H (1993). Wheezing and airflow obstruction during methacholine challenge in children with cystic fibrosis and in normal children. Am Rev Respir Dis.

[23] van Haren EH, Lammers JW, Festen J, van Herwaarden CL (1992). Bronchial vagal tone and responsiveness to histamine, exercise and bronchodilators in adult patients with cystic fibrosis. Eur Respir J.

[24] Balfour-Lynn IM, Welch K (2009). Inhaled corticosteroids for cystic fibrosis. Cochrane Database Syst Rev.

[25] Morgan WJ, Butler SM, Johnson CA, Colin AA, FitzSimmons SC (1999). Epidemiologic study of cystic fibrosis: design and implementation of a prospective, multicenter, observational study of patients with cystic fibrosis in the U.S. and Canada. Pediatr Pulmonol.

[26] McCuaig S, Martin JG (2013). How the airway smooth muscle in cystic fibrosis reacts in proinflammatory conditions: implications for airway hyper responsiveness and asthma in cystic fibrosis. Lancet Respir Med.

[27] Pepys MB, Hirschfield GM (2003). C-reactive protein: a critical update. J Clin Invest.

[28] Dahl M, Vestbo J, Lange P, Bojesen SE, Tybjaerg-Hansen A (2007). C-reactive protein as a predictor of prognosis in chronic obstructive pulmonary disease. Am J Respir Crit Care Med.

[29] Dahl M, Vestbo J, Zacho J, Lange P, Tybjaerg-Hansen A (2011). C reactive protein and chronic obstructive pulmonary disease: a Mendelian randomization approach. Thorax.

[30] Ngan DA, Wilcox PG, Aldaabil M, Li Y, Leipsic JA (2012). The relationship of systemic inflammation to prior hospitalization in adult patients with cystic fibrosis. BMC Pulm Med.

[31] Levy H, Kalish LA, Huntington I, Weller N, Gerard C (2007). Inflammatory markers of lung disease in adult patients with cystic fibrosis. Pediatr Pulmonol.

[32] Wolter J, Seeney S, Bell S, Bowler S, Masel P (2002). Effect of long term treatment with azithromycin on disease parameters in cystic fibrosis: a randomised trial. Thorax.

[33] Horsley AR, Davies JC, Gray RD, Macleod KA, Donovan J (2013). Changes in physiological, functional and structural markers of cystic fibrosis lung disease with treatment of a pulmonary exacerbation. Thorax.

[34] Ratjen F, Saiman L, Mayer-Hamblett N, Lands LC, Kloster M (2012). Effect of azithromycin on systemic markers of inflammation in patients with cystic fibrosis uninfected with Pseudomonas aeruginosa. Chest.

[35] Sullivan JC, Accurso FJ, Marigowda G, Beusmans J, Geho D (2016). Waltz. Improvement in inflammatory biomarkers in patients with cystic fibrosis homozygous for the F508del-CFTR mutation treated with lumacaftor and ivacaftor. J Cyst Fibros.

